# Supplementary material for: MetaRibo-Seq measures translation in microbiomes
Source: Nat Commun. 2020 Jun 29;11:3268. doi: 10.1038/s41467-020-17081-z (PMC7324362; doi:10.1038/s41467-020-17081-z)
Supplement: Supplementary file 10 — Supplementary Data 7 [file 41467_2020_17081_MOESM10_ESM.zip › File2/Confidence_High_Taxonomy/334545_out.krona.html]

Javascript must be enabled to view this page.

members
magnitude
magnitudeUnassigned
count
unassigned
taxon
rank

334545\_out

48

48
2
superkingdom

1239
phylum
48

186801
class
48

order
186802
48

48
186803
family

841
genus
48

species
166486

SRS014287\_contig\_number\_10060SRS014855\_contig\_number\_11288SRS015578\_contig\_number\_5143SRS015854\_contig\_number\_10992SRS015960\_contig\_number\_17208SRS017821\_contig\_number\_30662SRS018984\_contig\_number\_31814SRS019397\_contig\_number\_9677SRS022137\_contig\_number\_19141SRS024388\_contig\_number\_7606SRS024435\_contig\_number\_36251SRS043001\_contig\_number\_8804SRS043411\_contig\_number\_15542SRS043701\_contig\_number\_7720SRS050941\_contig\_number\_21373SRS052027\_contig\_number\_18977SRS053335\_contig\_number\_15139SRS055017\_contig\_number\_36636SRS056273\_contig\_number\_29662SRS056519\_contig\_number\_18485SRS058070\_contig\_number\_401SRS063518\_contig\_number\_39494SRS075341\_contig\_number\_24777SRS075716\_contig\_number\_3230SRS076756\_contig\_number\_19389SRS077454\_contig\_number\_15966SRS077641\_contig\_number\_9072SRS098827\_contig\_number\_12863SRS098881\_contig\_number\_24915SRS104084\_contig\_number\_11831SRS104975\_contig\_number\_8059SRS105153\_contig\_number\_39201SRS142503\_contig\_number\_51548SRS142781\_contig\_number\_11340SRS143070\_contig\_number\_17041SRS143417\_contig\_number\_25969SRS143598\_contig\_number\_23617SRS143722\_contig\_number\_11740SRS144362\_contig\_number\_29221SRS144537\_contig\_number\_18602SRS144714\_contig\_number\_13083SRS147271\_contig\_number\_25254SRS147919\_contig\_number\_2645SRS147977\_contig\_number\_5460SRS148091\_contig\_number\_4882SRS149244\_contig\_number\_9877SRS149879\_contig\_number\_contig-100\_8.146088SRS893378\_contig\_number\_18826
48
